# Synergistic Alcalase-papain hydrolysis of egg white protein: enhancing structural unfolding, antioxidant activity, and gastrointestinal stability

**DOI:** 10.1016/j.fochx.2026.104581

**Published:** 2026-10-08

**Authors:** Jialei Shi, Xianzhen Li, Xunyu Song, Siqi Wang

**Affiliations:** a School of Biological Engineering, Dalian Polytechnic University, Qinggongyuan 1, Dalian, Liaoning, China; b State Key Laboratory of Marine Food Processing and Safety Control, National Engineering Research Center of Seafood, School of Food Science and Technology, Dalian Polytechnic University, Dalian, Liaoning, China; c Dalian Key Laboratory for Precision Nutrition, Collaborative Innovation Center of Seafood Deep Processing, Academy of Food Interdisciplinary Science, Dalian Polytechnic University, Dalian, Liaoning, China; d Dalian Key Laboratory for Precision Nutrition, Dalian Polytechnic University, Dalian, Liaoning, China

**Keywords:** Enzymatic hydrolysis, Composite proteases, Bioactive peptides, Radical scavenging capacity

## Abstract

Egg white protein (EWP) is a promising source of bioactive peptides, but its compact structure limits enzymatic accessibility. This study compared single-protease hydrolysis using alcalase (Al) or papain (Pa) with composite alcalase-papain hydrolysis (Al—Pa) in terms of peptide production, structural modification, antioxidant activity, and digestive stability. Al—Pa achieved the highest hydrolysate yield (72.85%) and the largest proportion of peptides below 10 kDa (63.71%), including 26.34% below 3 kDa. Structurally, Al—Pa caused more extensive unfolding, hydrophobic group exposure, and microstructural reorganization than Al or Pa alone. During simulated gastrointestinal digestion, Al—Pa hydrolysates exhibited greater digestive stability, retained stronger DPPH radical-scavenging activity and high ABTS activity. The digested Al—Pa products also showed increases of 13.92% in branched-chain amino acids and 4.90% in hydrophobic amino acids, together with enrichment of sulfur-containing amino acids. These findings highlight the advantages of composite protease hydrolysis for producing EWP hydrolysates with sustained antioxidant functionality during digestion.

## Highlights

• Alcalas-papain hydrolysis increased peptide yield to 72.85%.
• Al—Pa produced 63.71% peptides below 10 kDa, including 26.34% below 3 kDa.
• Composite hydrolysis promoted extensive structural unfolding and rearrangement.
• Al—Pa hydrolysates retained strong antioxidant activity during digestion.

## Graphical abstract

**Figure:**
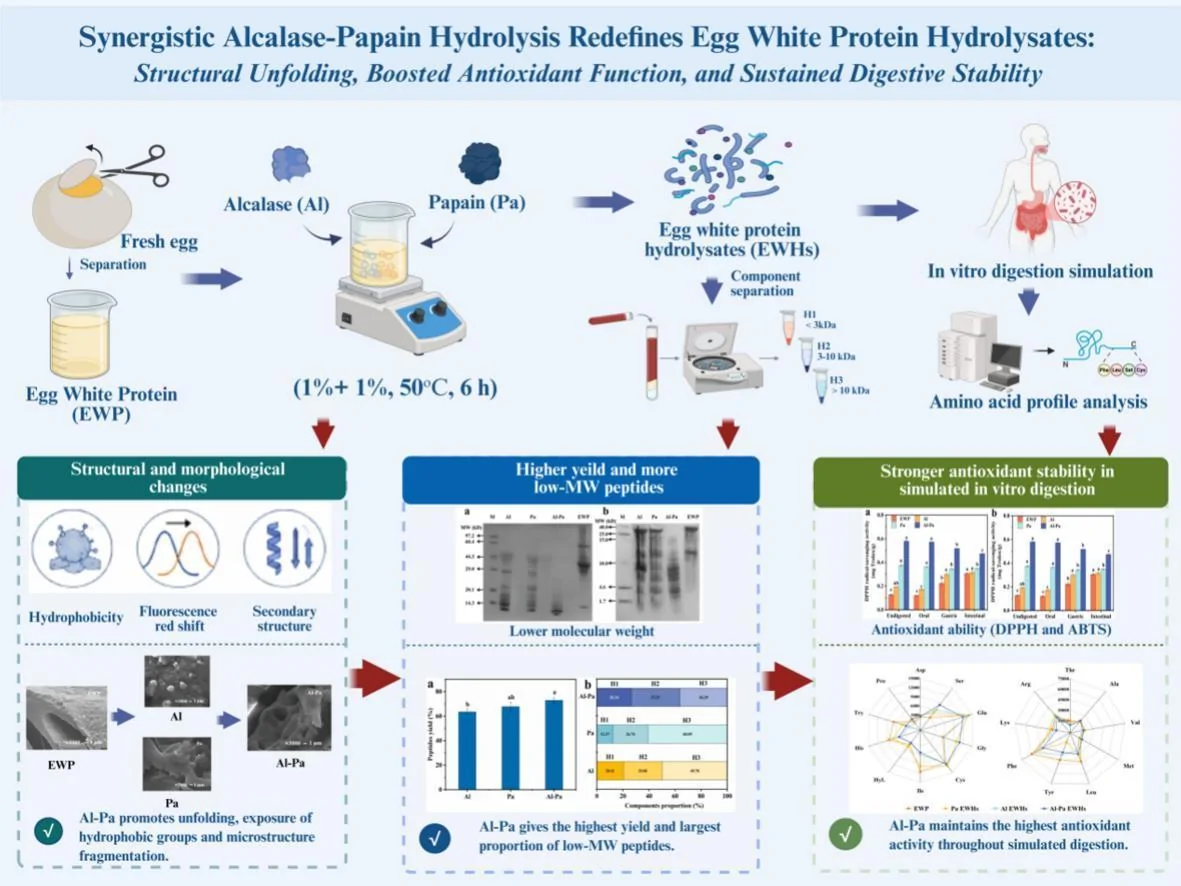

## 1 Introduction

Food-derived bioactive peptides have attracted substantial interest owing to their functions beyond basic nutrition, including antioxidant, antihypertensive, anti-inflammatory, and metabolic regulatory activities (Liu, Raynaldo, et al., 2024; Liu, Zhang, et al., 2024; Zhu et al., 2024). Such peptides have been generated from diverse proteins, and their biological performance is strongly influenced by amino acid composition and peptide size (Manfredini et al., 2021; Mora & Toldrá, 2023). Egg white protein (EWP) is a high-quality and abundant protein source and represents an attractive precursor for bioactive peptide production. However, native EWP contains compact, highly organized protein structures that restrict protease accessibility and limit the efficient release of low-molecular-weight peptides (Habinshuti et al., 2023). Given these structural constraints, developing an efficient hydrolysis strategy for EWP that enhances peptide yield and mitigates activity loss during prolonged gastrointestinal digestion is essential.

Enzymatic hydrolysis is widely used to release bioactive peptides from proteins, however, hydrolysis efficiency and the resulting bioactivity depend strongly on protease specificity and substrate accessibility (Mora & Toldrá, 2023). Currently, various proteases have mature production processes, among which alcalase and papain have been widely applied due to their high efficiency and safety. However, a single protease is limited by substrate recognition and accessibility, making it difficult to achieve the expected functional effect (Yao et al., 2026). This limitation can be overcome by composite protease systems that combine different catalytic and cleavage preferences. By targeting a broader range of peptide bonds, composite protease can enhance the hydrolysis efficiency and facilitate the release of specific amino acids, which are the key components of functional specificity (Manfredini et al., 2021). Several studies have demonstrated the advantages of using composite proteases to enhance protein hydrolysis efficiency and hydrolysates biological activity (Aguilar et al., 2019; Yan et al., 2022). A representative case is the composite treatment with alcalase and papain, which increased both peptide content and antioxidant activity in chicken breast protein (Setthaya et al., 2021). Nevertheless, whether this composite strategy offers similar advantages for EWP, and how the resulting structural and peptide-profile changes influence functionality during gastrointestinal digestion, remain unclear.

The objective of this study was to evaluate the effects of single-protease hydrolysis on egg white protein (EWP) in comparison with the composite Al—Pa hydrolysis strategy. The selection of these two proteases was based on their complementary specificities: Al is a serine endoprotease with relatively narrow cleavage specificity, whereas Pa is a cysteine protease exhibiting broader specificity. The resulting hydrolysates were elucidated in terms of yield, degree of hydrolysis and molecular-weight distribution. Structural difference of hydrolysates were identified using Fourier-transform infrared spectroscopy (FTIR), intrinsic fluorescence spectroscopy, and scanning electron microscopy (SEM). In addition, the dynamic changes in antioxidant activity during simulated prolonged gastrointestinal digestion were investigated, along with the amino acid composition and functional characteristics of the digesta. These findings provide mechanistic insights into the efficient production of bioactive EWHs and support the potential application of functional food ingredients with enhanced digestive stability and antioxidant efficacy, particularly for elderly and patient populations.

## 2 Materials and methods

### 2.1 Materials

Fresh eggs were purchased from a local supermarket in Dalian, Liaoning Province, China. Alcalase was purchased from Novo Co., Ltd. (Beijing, China) and papain was provided by Yuanye Biotechnology Co., Ltd. (Shanghai, China). All chemical reagents used were of analytical grade.

### 2.2 EWP and EWHs preparation

Egg yolks were carefully separated, and the egg whites were collected and homogenized for 5 min. The protein concentration of the homogenized EWP solution used for hydrolysis was adjusted to 10 mg/mL with deionized water. The EWP solution was preheated at 55 °C for 30 min and then subjected to enzymatic hydrolysis. For the single-protease treatments, alcalase (Al) or papain (Pa) was added at 2% (*w*/w), and hydrolysis was conducted at 50 °C for 6 h. For the composite treatment (Al—Pa), 1% (*w*/w) Al was added and incubated at 50 °C for 3 h, followed by 1% (*w*/w) Pa under the same conditions for an additional 3 h. After hydrolysis, the mixture was heated in an 80 °C water bath for 20 min to inactivate the proteases. The sample was then cooled to room temperature and centrifuged at 6000 rpm for 20 min. The resulting supernatant, designated as egg white protein hydrolysates (EWHs), was collected for further analysis. For the control group (EWP), identical processing were applied and the protease solution was replaced with deionized water.

### 2.3 Yield and peptide component determination

The yield of the EWHs was calculated as the ratio of the protein recovered in the hydrolysate supernatant to the protein content of the initial EWP, according to the Kjeldahl method as described by Aguirre (2023). Subsequently, the EWH samples were subjected to sequential ultrafiltration using dialysis membranes with the MW cut-offs of 10 kDa and 3 kDa, and quantitative analysis of each component was conducted according to the method of Cao et al. (2024).

### 2.4 Molecular weight (MW) and particle size distribution

The MW of EWP and EWHs was determined using electrophoresis analysis. For sodium dodecyl sulfate-polyacrylamide gel electrophoresis (SDS-PAGE), samples were mixed with SDS loading buffer, boiled for 5 min, and 10 μL of each sample was loaded per lane. A 5% concentrated gel and 12% separation gel were prepared, and the gels were concentrated at 80 V and separated at 120 V. Low-MW components were further analyzed by Tricine-SDS-PAGE according to Berlian et al. (2023). Three-layer SDS gel was gradiently set as 4–10-16.5%, and proteins were separated at 120 V for 120 min.

Particle size and zeta potential were determined using a Malvern Zeta Plus analyzer (Malvern Panalytical Ltd., UK) at 25 °C, with samples dispersed in deionized water at 1.0 mg/mL, following the method of Zhang, Cui, et al. (2025), Zhang, Liu, et al. (2025) and Zhang, Zang, et al. (2025). The zeta potential was measured using a DTS1070 cell, and particle size was determined using a DTS0012 cell, with a refractive index set at 1.5, an equilibration time of 120 s.

### 2.5 Intrinsic fluorescence and surface hydrophobicity

Intrinsic fluorescence was recorded using a fluorescence spectrophotometer (F-4600, Hitachi, Japan) following the method proposed by Li et al. (2024). EWP and EWHs were dissolved in 10 mmol/L phosphate buffer solution (PBS, pH 7.2) to a final concentration of 1 mg/mL. The fluorescence spectra were recorded at an excitation wavelength of 290 nm, with a scan rate of 120 nm/min and a slit width of 5 nm.

Surface hydrophobicity (S_0_) was determined using 1-anilino-8-naphthalene sulfonate (ANS) as an exogenous fluorescence probe (Wang et al., 2020). A mixture of 1 mL sample and 20 μL 8 mmol/L ANS solution (0.1 mol/L PBS, pH 7.2) was vortexed for 30 s and subsequently incubated in the dark at room temperature for 15 min. Fluorescence intensity was recorded using the F-4600 spectrophotometer at excitation and emission wavelengths of 365 and 484 nm, respectively. A relationship curve was constructed by plotting fluorescence intensity against sample concentration, and the slope of this curve was used to quantify S_0_.

### 2.6 Fourier transform infrared (FT-IR) spectroscopy

The FT-IR spectra of EWP and EWHs were acquired using a FT-IR spectrometer (Spectrum Two, PerkinElmer, USA). The freeze-dried sample was thoroughly ground with potassium bromide at a ratio of 1:100 (*w*/w) and compressed into a semi-transparent pellet. Spectral scanning was performed over a range of 4000–400 cm^−1^. The amide I region (1600–1700 cm^−1^) was subjected to Fourier self-deconvolution and Gaussian curve fitting using PeakFit 4.12 (Systat Software Inc., USA) to estimate the secondary structure components (Asaithambi et al., 2022).

### 2.7 Scanning electron microscopy (SEM)

The surface morphology of EWP and EWHs was examined a scanning electron microscope (S3400, Hitachi, Japan). Freeze-dried samples were mounted on specimen stubs using conductive double-sided adhesive tape and sputter-coated with an approximately 5 nm thick layer of gold. Then, the surface structure of the samples was observed under an accelerating voltage of 20 kV.

### 2.8 In vitro simulated gastrointestinal digestion and functional characterization

#### 2.8.1 Simulated in vitro digestive products preparation

A modified INFOGEST 2.0 protocol with prolonged digestion times to simulate the digestive process in populations such as the elderly or patients (Brodkorb et al., 2019; Wang, Wang, et al., 2022; Wang, Yang, et al., 2022; Wang, Zhao, et al., 2022; da Silvaa et al., 2023). EWP and EWHs were mixed with simulated saliva (pH 7.0) at a ratio of 1:1 (*w*/*v*) and incubated at 37 °C for 2 min, shaking at 100 rpm. Subsequently, simulated gastric fluid (pH 3.0) was then added in the mixture at a 1:1 (*v*/v) ratio, and incubated at 37 °C for 6 h. The resulting gastric digesta were then mixed with simulated intestinal fluid at a 1:1 (*v*/v) ratio and incubated at 37 °C (pH 7.0) with 100 rpm for an additional 6 h. The enzymatic activity was terminated by heating the reaction mixture in boiling water for 20 min. The digesta from each in vitro digestion stage was collected for further analysis.

#### 2.8.2 Apparent digestibility (AD)

The AD of the sample was determined based on the content of free amino groups according to the method described by Yan et al. (2022). Briefly, 400 μL of each sample was mixed with 3 mL o-phthalaldehyde reagent, homogenized and incubated at 37 °C for 2 min. Absorbance was measured at 340 nm using a microplate reader (F50, TECAN, Switzerland), and serine solution was used as the standard reference.

#### 2.8.3 Antioxidant activity determination

The 2,2-Diphenyl-1-picrylhydrazyl (DPPH) radical scavenging activity was evaluated according to Leiva-Portilla et al. (2023). The sample was prepared into 2.0 mg/mL and mixed with 2 mL of 0.2 mmol/L DPPH solution. After incubation in the dark for 30 min, the absorbance was measured at 517 nm using an ultraviolet-visible spectrometer (Lambda 35, PerkinElmer, USA).

The 2,2′-Azinobis (3-ethylbenzothiazoline-6-sulfonic acid) (ABTS) radical scavenging activity was assessed according to the method of Gulcin and Alwasel (2023) with some modifications. 7.4 mmol/L ABTS^+^ solution was prepared with 2.6 mmol/L potassium persulfate, placed in the dark at room temperature for 15 h before use. 100 μL sample was added to 3.5 mL ABTS^+^, stood for 30 min in the dark. The absorbance was measured at 734 nm, and deionized water was used to replace the sample as the blank.

#### 2.8.4 Amino acid composition analysis

The amino acid composition of the digesta was analyzed according to the method of Tian et al. (2022) with slight modifications. Briefly, the EWP and EWHs digest were hydrolyzed with 6 mol/L HCl at 110 °C for 22 h. The total amino acid composition was determined using an automatic amino acid analyzer (LA8080, Hitachi, Japan), with quantification performed using the external standard method.

### 2.9 Statistical analysis

All the measurements were conducted in three separate replicates, and the results were expressed as the mean ± standard deviation (SD). Statistical analysis was performed with one-way analysis of variance at *p* < 0.05 using SPSS 24.0 (SPSS Inc., USA). The images were drawn using Origin 2024 (Origin Lab Co., USA).

## 3 Results and discussion

### 3.1 Yield and molecular weight (MW) of EWHs

#### 3.1.1 Electrophoretic analysis

MW serves as a critical indicator for evaluating the hydrolysis hydrolysis degree of protein hydrolysates (Chang et al., 2025). SDS-PAGE and Tricine-SDS-PAGE were used to compare the hydrolysate derived from EWP subjected to Al, Pa, or composite Al—Pa hydrolysis (Fig. 1). Untreated EWP displayed prominent protein bands in the range of 29.0–64.4 kDa, along with a faint band below 14.3 kDa, corresponding to ovalbumin and lysozyme, respectively (Désert et al., 2001). In contrast, all EWHs showed hydrolysis at the 44.3 kDa band and produced distinct new bands below 29 kDa. Further insights were obtained from Tricine-SDS-PAGE, which revealed that the composite protease generated EWHs with markedly lower MW compared to those produced by single proteases, resulting in the products mainly consisting of peptide segments with MW lower than 10 kDa. Notably, Al demonstrated stronger enzymatic hydrolysis ability than Pa, and their synergistic application further enhanced the hydrolysis degree, yielding a substantial fraction of peptides with reduced MW below 1.7 kDa. These results indicate that the composite treatment cleaved EWP more efficiently than either single protease treatment in EWP hydrolysis. Mechanistically, the initial Al treatment disrupts accessible regions of EWP and loosens the native structure, thereby exposing additional peptide bonds to Pa in the subsequent hydrolysis process. Consequently, this sequential action effectively broadened the range of cleavage sites and favored the production of shorter peptide fragments (Akimova et al., 2024; Shukla et al., 2022). Overall, these results demonstrate that the combined use of Al and Pa is an effective strategy for generating low-MW bioactive peptides from EWP.

**Fig. 1:**
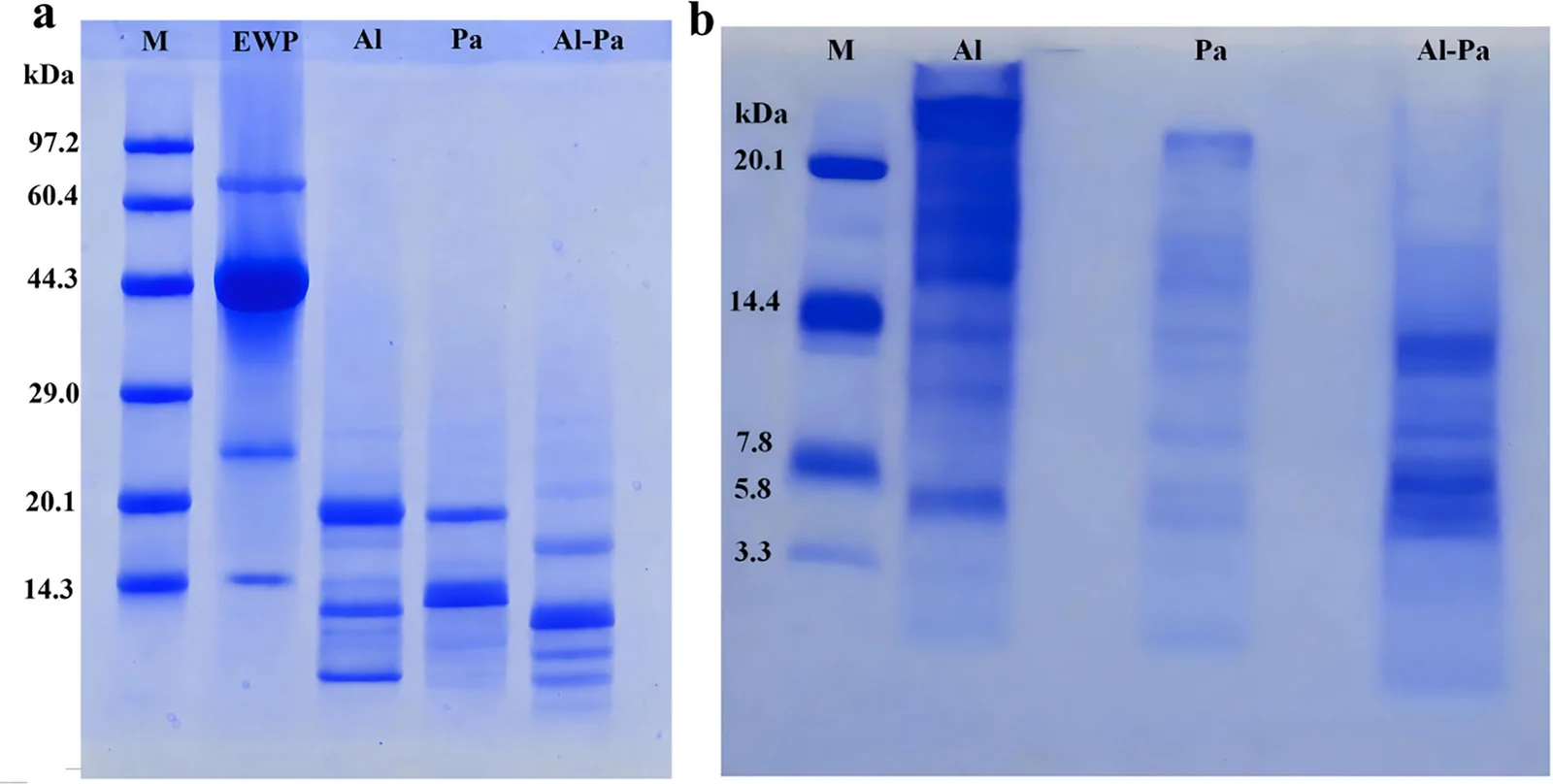
Gel electrophoresis analysis of EWP and EWHs (a) SDS-PAGE and (b) Tricine-SDS-PAGE. Abbreviation: Al is single-alcalase treatment; Pa is single-papain treatment; Al—Pa is composite alcalase and papain treatment.

#### 3.1.2 Yield and MW distribution of EWHs

The yield and MW distribution of EWHs further confirmed the advantage of the composite treatment (Fig. 2). Compared with single protease treatments, the combined protease treatment (Al—Pa) resulted in a higher yield and produced a greater proportion of low-MW components. Specifically, the EWHs yield reached a maximum of 72.85% with Al—Pa, whereas the yields of Pa and Al were 67.43% and 63.77%, respectively (Fig. 2a). Based on MW, the hydrolysates were separated into three fractions, H1 was the fraction below 3 kDa, H2 was the 3–10 kDa fraction, and H3 was the fraction over 10 kDa. Untreated EWP was predominantly consisted of proteins larger than 10 kDa, consistent with previous study (Lyu et al., 2023). Following enzymatic treatment, the total proportion of hydrolysates below 10 kDa (H1 and H2) increased markedly, reaching the highest level for Al—Pa (63.71%), followed by Al (50.22%) and Pa (39.11%) (Fig. 2b). These results demonstrate that Al—Pa increased total hydrolysate recovery, and further extended the proportion of low-MW products (<10 kDa). Low-MW peptides frequently show stronger antioxidant potential because smaller peptides effectively expose redox-active residues and may interact more readily with radical species (Chen et al., 2023; Li et al., 2025; Wang et al., 2023). Overall, the yield and MW distribution results support a synergy strategy in which the Al treatment increases substrate accessibility, and the Pa treatment further cleaves newly exposed peptide bonds, improving both solubilization and peptide-size reduction. The synergistic action of Al and Pa resulted in a higher degree of hydrolysis, corroborating previous studies on the enzymatic modification of EWP for improved functional properties (Lyu et al., 2023; Tang et al., 2019).

**Fig. 2:**
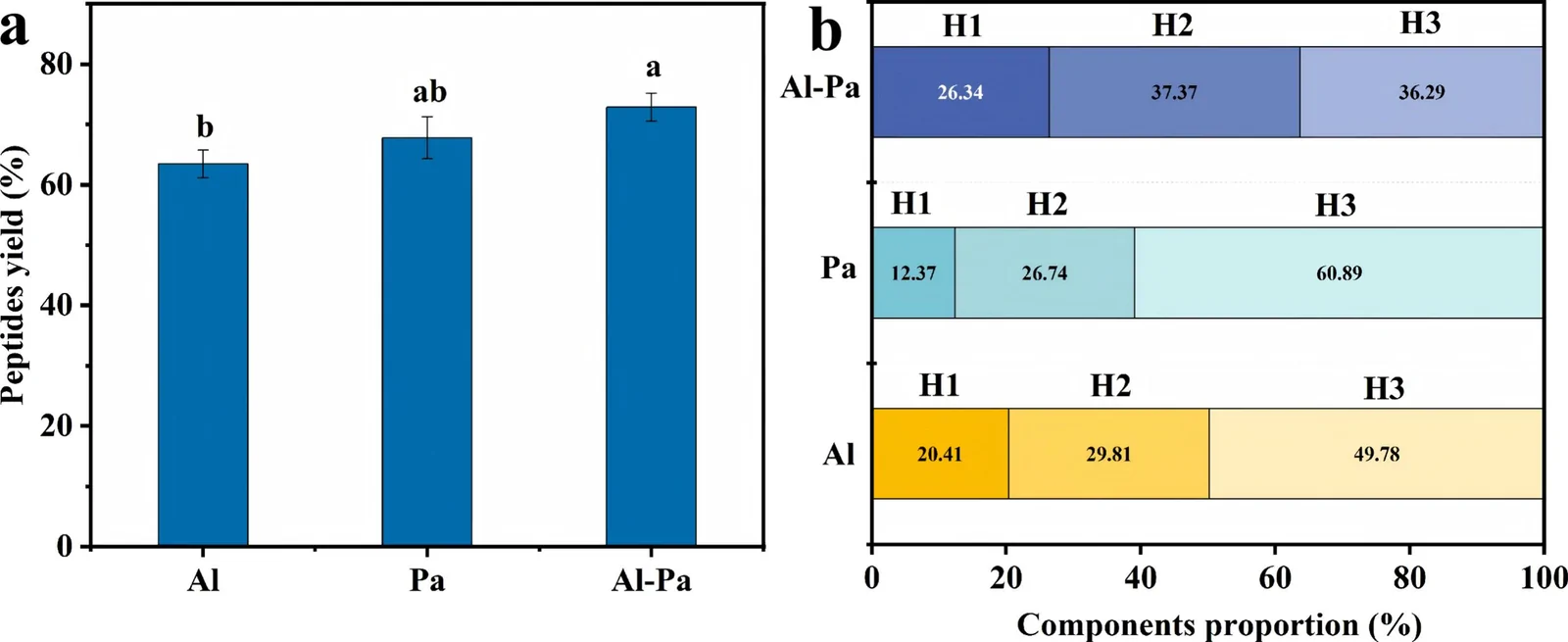
(a) The yield of enzymatically hydrolyzed peptides and (b) the molecular weight distribution of EWHs. Abbreviation: Al is single-alcalase treatment; Pa is single-papain treatment; Al—Pa is composite alcalase and papain treatment. Different lowercase letters indicate significant differences between groups (*n* = 3, *p* < 0.05).

### 3.2 Structural properties analysis

#### 3.2.1 Particle size distribution and zeta potential analysis

The particle size distribution and zeta potential of EWP and EWHs are presented in Fig. 3. Untreated EWP exhibited a particle size distribution ranging from 10 to 100 nm, suggesting a relatively small hydrodynamic size distribution, along with a zeta potential of approximately −12 mV. Following enzymatic hydrolysis, the particle size of EWHs increased with the Al—Pa treatment showing a more pronounced increase in particle size compared to single protease hydrolysates (Fig. 3a). Proteolysis exposes hydrophobic residues that were previously buried within the native protein. These newly exposed nonpolar surfaces reduce unfavorable contact with water, therefore promoting peptide-peptide aggregation. This tendency is further favored when electrostatic repulsion is weakened, providing a thermodynamic basis for the formation of larger hydrodynamic assemblies despite the lower molecular weight of the constituent peptides (Cui et al., 2021; Li et al., 2024).

**Fig. 3:**
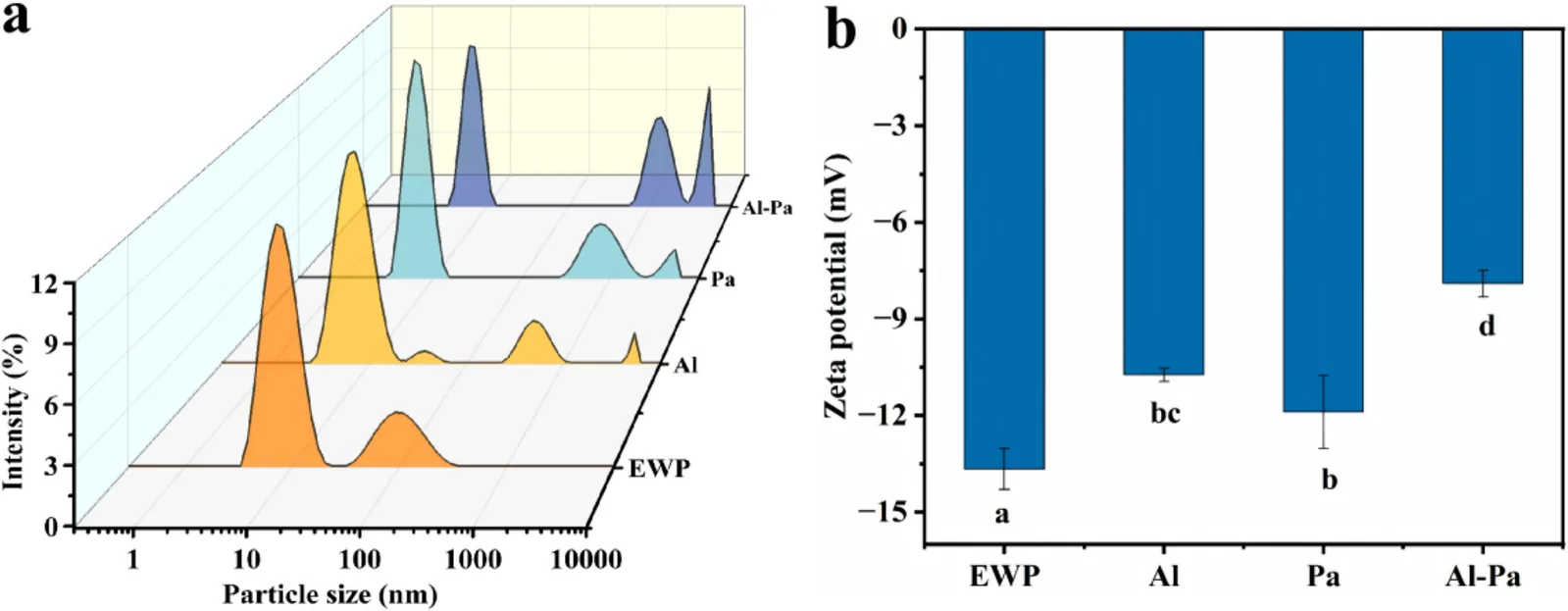
(a) The particle sizes and (b) zeta potential of EWP and EWHs. Abbreviation: Al is single-alcalase treatment; Pa is single-papain treatment; Al—Pa is composite alcalase and papain treatment. Different lowercase letters indicate significant differences between groups (*n* = 3, *p* < 0.05).

Zeta potential quantifies the surface charge of protein molecules, with a higher absolute value indicating stronger electrostatic repulsion and thus enhanced colloidal stability of the system (Guo et al., 2024). Following enzymatic hydrolysis, all EWH samples exhibited a decrease in the absolute zeta potential compared to native EWP, with the most pronounced reduction observed in the Al—Pa treated sample (Fig. 3b). This reduction is primarily attributed to the enhanced electrostatic screening caused by hydrolysis-induced compositional changes. As a result, stronger short-range attractive interactions and a greater tendency for aggregation are promoted (Ai et al., 2019). Additionally, the disruption of the balance between electrostatic repulsion and van der Waals attraction may promote irreversible aggregation (Li et al., 2024). The weakened electrostatic interactions provide a plausible explanation for the increased particle size observed after hydrolysis, which is consistent with previous findings (Shi et al., 2024).

#### 3.2.2 Surface hydrophobicity and intrinsic fluorescence

Surface hydrophobicity (S_0_), which reflects the number of exposed non-polar groups on the protein surface (Tu et al., 2024), serves as a major driving force for maintaining tertiary structure (Almeida et al., 2021). As shown in Fig. 4a, enzymatic hydrolysis significantly increased the S_0_ of EWHs compared to untreated EWP (*p* < 0.05). Among the hydrolysates, the Al—Pa hydrolysates exhibited the highest S_0_, which strongly correlated with the degree of hydrolysis (Liu, Raynaldo, et al., 2024; Liu, Zhang, et al., 2024). This observation aligns with the findings of Wang et al. (2023), which reported a similar relationship between hydrolysis degree and S_0_ in sturgeon protein hydrolysates. In native EWP, hydrophobic groups and helical structures are predominantly buried in the protein interior, while polar residues are exposed to the surface. Enzymatic hydrolysis disrupted this spatial structure of EWP, causing the polypeptide chain to unfold and exposing previously hidden hydrophobic regions, thereby resulting in an increase in S_0_ (Lyu et al., 2023). This structural rearrangement facilitates intermolecular interactions and may promote the formation of larger aggregates. Furthermore, the synergistic action of the composite protease (Al—Pa) generated a higher proportion of low-molecular-weight peptides with more hydrophobic amino acids.

**Fig. 4:**
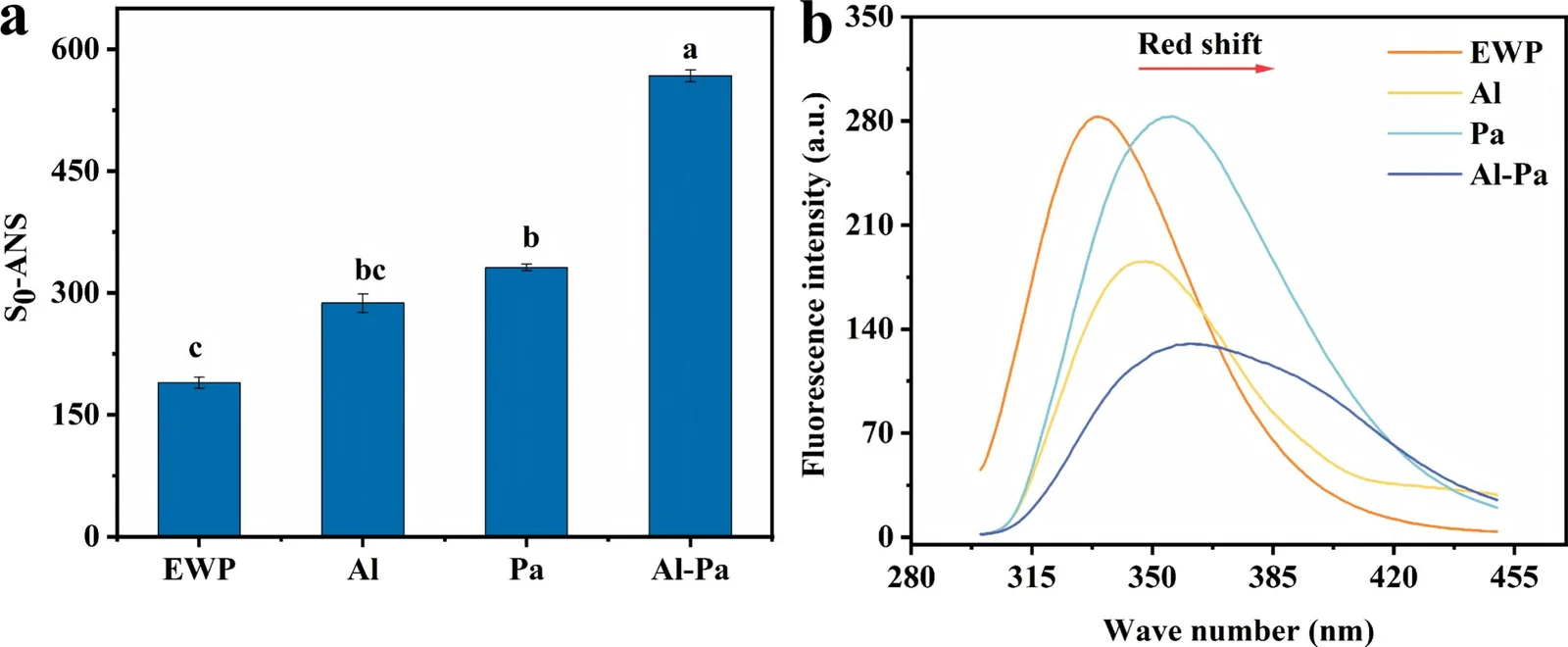
(a) The surface hydrophobicity and (b) intrinsic fluorescence spectroscopy of EWP and EWHs. Abbreviation: Al is single-alcalase treatment; Pa is single-papain treatment; Al—Pa is composite alcalase and papain treatment. Different lowercase letters indicate significant differences between groups (*n* = 3, *p* < 0.05).

Intrinsic fluorescence spectroscopy was employed to elucidate structural alterations in EWP and EWHs, as depicted in Fig. 4b. The aromatic amino acids in EWP and EWHs, tryptophan (Trp), tyrosine (Tyr), and phenylalanine (Phe), serve as intrinsic fluorescence probes in investigating the microenvironment and conformational dynamics (Kyrychenko & Ladokhin, 2024). Native EWP exhibited a fluorescence emission maximum at 334 nm, indicating a folded conformation in which aromatic residues are predominantly buried within the hydrophobic core of the protein (Akbari et al., 2020). Following enzymatic hydrolysis, all EWHs displayed a red shift in the maximum emission wavelength along with a reduction in fluorescence intensity. The most pronounced changes were observed in the Al—Pa treated sample, which showed a shift to 361 nm and the most substantial decrease in fluorescence intensity. This phenomenon indicates that hydrolysis products with exposured fluorophores residues to a more polar solvent environment. Proteolysis disrupts the native protein structure and induces hydrolysis of peptide bonds, resulting in structural expansion and increased solvent accessibility. Meanwhile, exposure of Trp to aqueous solvents results in fluorescence quenching due to increased collisional frequency and interactions with protons (Bagheri et al., 2024). These findings are consistent with previous reports on hydrolyzed walnut protein, where similar red shifts were observed, further confirming that proteolysis leads to the exposure of hydrophobic groups (Moghadam et al., 2020). Together with the increase in S_0_, these results support more extensive structural unfolding and rearrangement in the Al—Pa hydrolysates than in the single-enzyme products.

#### 3.2.3 FT-IR and secondary structure analysis

The conformational changes in EWP following enzymatic hydrolysis were examined by FT-IR spectroscopy. As shown in Fig. 5, all hydrolysates displayed alterations in their secondary structure compared to the native EWP. The spectral profiles were consistent with those of ovalbumin, the major component of egg white, which shares high structural homology with EWP (Liu et al., 2018). The amide absorption region at 3500–3000 cm^−1^, attributed to N—H and O—H stretching vibrations, reflects the hydrogen bonding network within the polypeptide backbone. Additionally, the region from 3000 to 2800 cm^−1^, corresponding to symmetric and asymmetric C—H stretching vibrations of aliphatic groups. Changes in the position or intensity of these regions following hydrolysis therefore reflect alterations in hydrogen-bonding environments and the exposure or reorganization of side chains, rather than the formation of a new covalent complex (Liu et al., 2026). A notable reduction in absorption intensity was observed in the 3000–2800 cm^−1^ region for all EWHs, indicating that enzymatic hydrolysis disrupts hydrophobic domains and compromises hydrogen bonding essential for maintaining secondary structure (Hubbard & Haider, 2010). Notably, the amide I region (approximately 1700–1600 cm^−1^), dominated by peptide-backbone C=O stretching, is particularly sensitive to protein secondary structure. The spectral changes observed among Al, Pa, and Al—Pa indicate that cleavage of peptide bonds reorganized both backbone interactions and the local environment of functional groups. The more pronounced changes in Al—Pa are consistent with its greater proteolytic disruption and hydrophobic exposure. These alterations confirm that proteolytic treatment significantly affects the structural integrity of EWP.

**Fig. 5:**
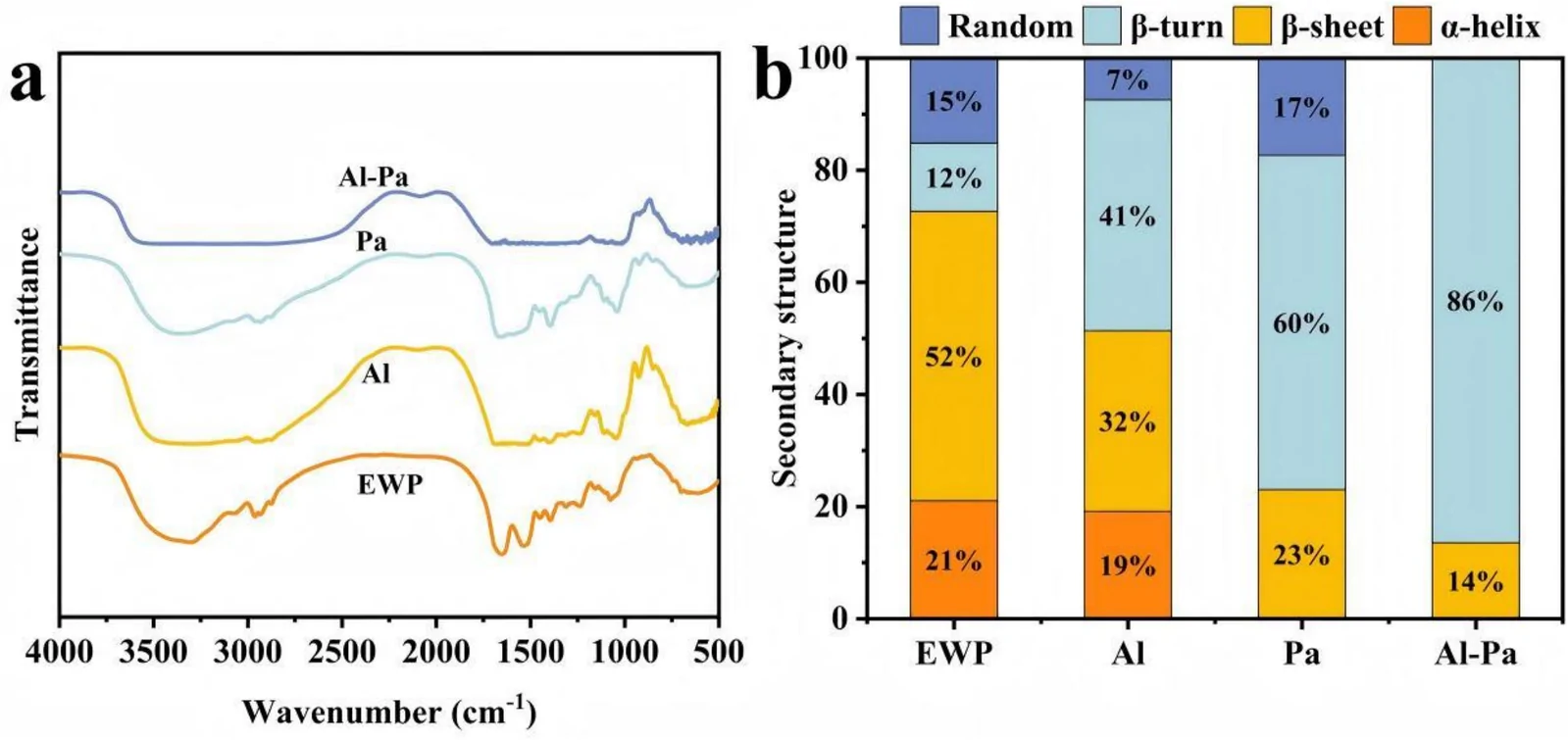
(a) The FTIR spectra and (b) secondary structure composition of EWP and EWHs. Abbreviation: Al is single-alcalase treatment; Pa is single-papain treatment; Al—Pa is composite alcalase and papain treatment.

The amide I band was deconvoluted to estimate the relative proportions of α-helix, β-sheet, β-turn, and random-coil structures (Zhang, Liu, et al., 2025). The characteristic peaks of α-helix, β-sheet, β-turn, and random coil in the amide I band were located at 1650–1660 cm^−1^, 1610–1640 cm^−1^, 1660–1665 cm^−1^, and 1640–1650 cm^−1^, respectively. Native EWP was fitted as 21.05% α-helix, 51.60% β-sheet, 12.18% β-turn, and 15.17% random coil, whereas hydrolysis markedly altered the fitted secondary-structure distribution (Fig. 5b). The most pronounced change was observed in Al—Pa, for which the β-turn-associated component accounted for the dominant fitted fraction (86.44%), while α-helical and random-coil components became very small or undetectable within the applied fitting model. This distinct behavior may be attributed to strong hydrophobic interactions, particularly those involving Phe and Pro residues, which could promote partial structural refolding (Gupta & Uversky, 2024). Collectively, the comprehensive evidence obtained from FT-IR combined with multiple structural analyses indicates that Al—Pa caused the most extensive conformational disruption among the treatments. These findings highlight the critical role of composite enzymatic hydrolysis in modulating the secondary structure of proteins, which ultimately shapes functional properties through the exposure of functional groups and reorganization of critical molecular interactions.

#### 3.2.4 The surface morphology analysis of EWP and EWHs

SEM was employed to examine the microstructure of EWP and EWHs at magnifications of 500× and 5000×, in order to elucidate the effects of different enzymatic treatments (Fig. 6). Enzymatic hydrolysis with either Al or Pa resulted in the transformation of these thick blocks into thinner sheet-like structure, accompanied by surface fragmentation and the appearance of rough, multi-layered particulate aggregates. In contrast, the EWHs produced by Al—Pa displayed a distinctly globular morphology, more pronounced than that of the single-enzyme treatments. The synergistic action of the composite protease intensified the hydrolysis process, leading to a collapsed protein structure characterized by smooth edges and surface protrusions, which suggests more extensive proteolytic degradation (Akimova et al., 2024). Notably, the Al—Pa treatment resulted in the formation of conspicuous cavities, a phenomenon not observed in the single-protease hydrolysates. The presence of clustered particulate features is consistent with the post-hydrolysis aggregation phenomenon revealed by particle size analysis. These structural alterations are consistent with previous observations in rice bran globulin following trypsin hydrolysis (Wang, Wang, et al., 2022; Wang, Yang, et al., 2022; Wang, Zhao, et al., 2022).

**Fig. 6:**
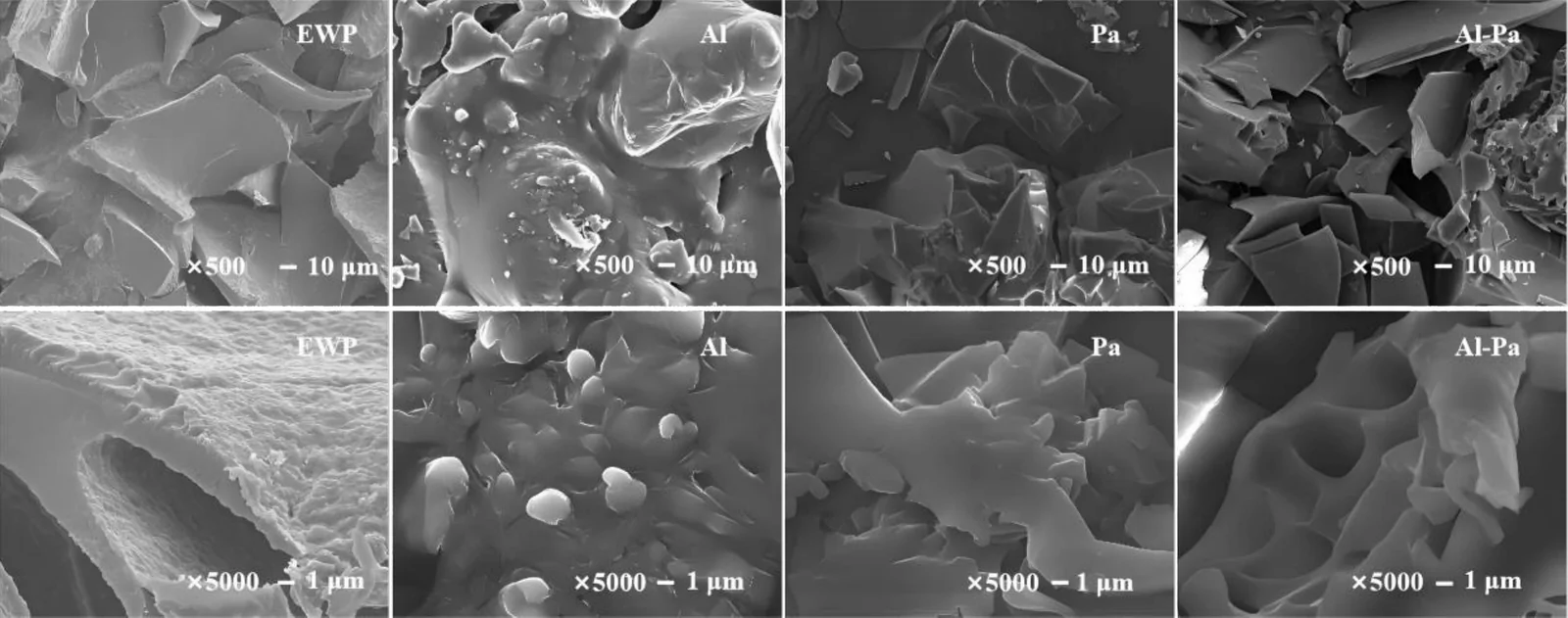
Scanning electron microscope images of different enzymatic hydrolysis products. Abbreviation: Al is single-alcalase treatment; Pa is single-papain treatment; Al—Pa is composite alcalase and papain treatment.

### 3.3 Performance changes during the in vitro digestion process

#### 3.3.1 The apparent digestibility (AD) during the digestion process

The AD is expressed by the determination of free amino groups in the samples during the digestion process. Before gastrointestinal digestion, the Al-, Pa-, and Al-Pa-treated samples were already hydrolysates produced, whereas EWP served as the non-protease-treated control. Before the simulated digestion, the Al—Pa showed a higher initial level of free amino groups of 78.64% (undigested) than those produced by the single proteases Al or Pa, demonstrating superior efficiency of composite protease in cleaving peptide bonds (Fig. 7). This finding is consistent with previous reports indicating that composite proteases enhance hydrolysis by accessing a broader range of cleavage sites, thereby promoting proteolytic efficacy and reducing MW of the resulting hydrolysates (Naveed et al., 2021). The most pronounced increase was observed at the gastric and intestinal stages, which can be attributed to the synergistic effects of low pH and the action of digestive enzymes such as pepsin and trypsin. Notably, all samples eventually reached comparable final AD values, indicating that digestion proceeded to completion and providing a consistent foundation for subsequent analysis of the digested products.

**Fig. 7:**
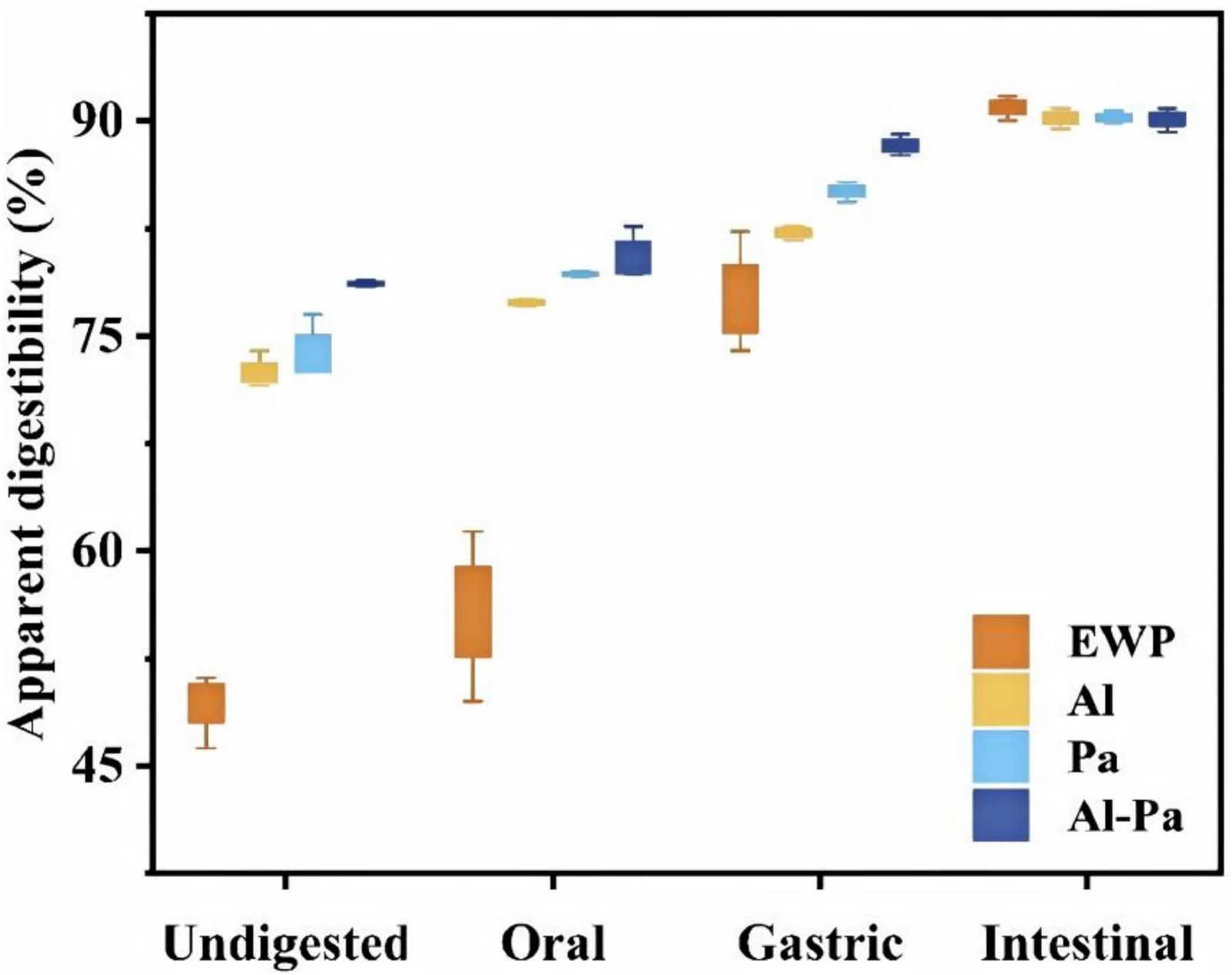
The changes in hydrolysis degree during the digestion process. Abbreviation: Al is single-alcalase treatment; Pa is single-papain treatment; Al—Pa is composite alcalase and papain treatment.

#### 3.3.2 The stability of antioxidant capacity during the digestion process

The DPPH and ABTS radical scavenging assays are widely used methods to evaluate the antioxidant capacity of protein hydrolysates. As shown in Fig. 8a, the DPPH assay indicated that EWHs prepared with Al—Pa consistently exhibited the highest radical scavenging activity across all digestion stages, followed by those treated with Pa, Al, and untreated EWP. Although the absolute DPPH response changed during digestion, the relative advantage of Al—Pa was maintained, indicating that the antioxidant functionality generated by composite hydrolysis was not eliminated by further digestive proteolysis. It should be noted that the high-concentration ethanol solution utilized in the DPPH assay may provoke protein aggregation, potentially resulting in an underestimation of the true radical scavenging activity. In the ABTS assay, Al- and Al-Pa-derived samples remained among the strongest scavengers throughout digestion. Their high activity arises from the increased exposure of electron-donating groups, including sulfur-containing residues and tyrosine-associated phenolic groups. It is also reflects the greater accessibility of hydrophobic peptide regions to free radicals (Fu et al., 2023; Zhu et al., 2024). Overall, the antioxidant results support functional persistence rather than a simple monotonic increase in activity during digestion.

**Fig. 8:**
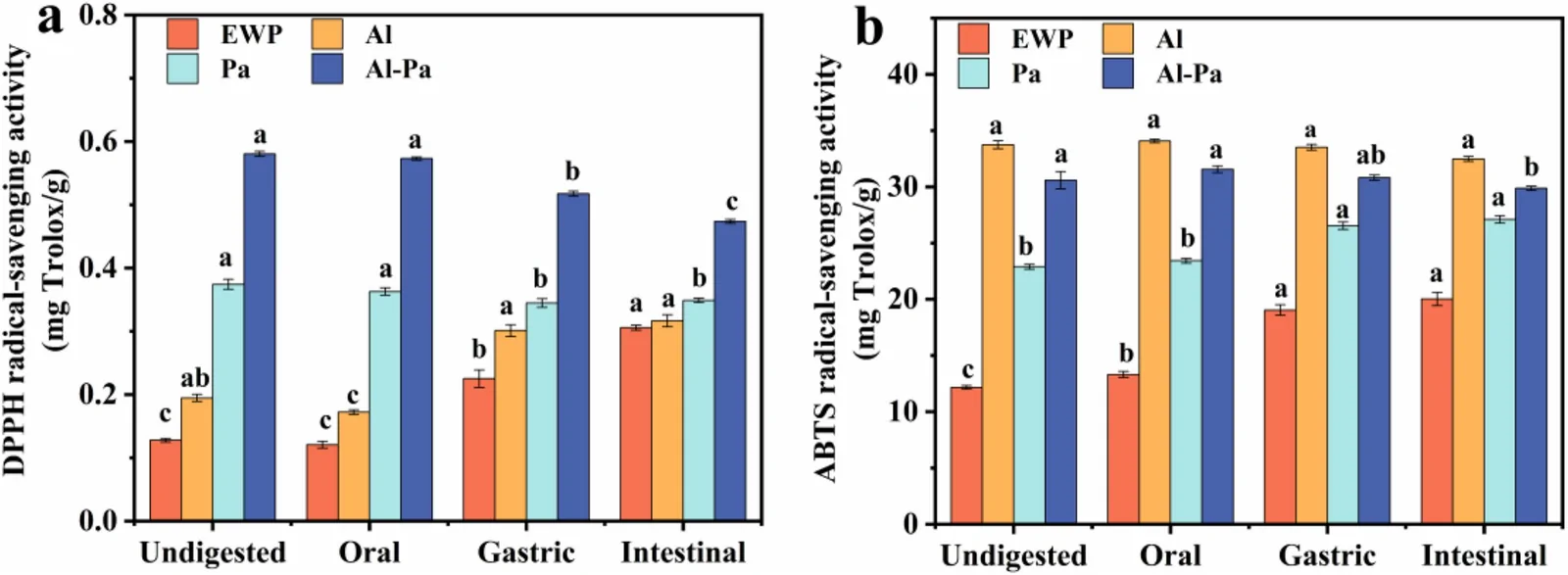
The analysis of antioxidant capacity during in vitro digestion: (a) DPPH free radical scavenging ability and (b) ABTS free radical scavenging ability. Abbreviation: Al is single-alcalase treatment; Pa is single-papain treatment; Al—Pa is composite alcalase and papain treatment. Different lowercase letters indicate significant differences between groups (*n* = 3, *p* < 0.05).

#### 3.3.3 The amino acid components of the digestive products

To characterize the compositional features of the final gastrointestinal digesta, amino acid profiles were examined (Fig. 9 and Table 1). Compared with the EWP digest, the enzyme-treated digesta generally showed higher levels of alanine (Ala), isoleucine (Ile), cysteine (Cys), tyrosine (Tyr), glycine (Gly), methionine (Met), histidine (His), and leucine (Leu), whereas lower levels were observed in threonine (Thr), serine (Ser), glutamic acid (Glu), phenylalanine (Phe), lysine (Lys), and tryptophan (Trp) (Fig. 9). Al—Pa showed the highest mean concentrations of branched-chain amino acids (BCAAs; 79.41 ng/mL) and hydrophobic amino acids (HAAs; 183.61 ng/mL), corresponding to increases of 13.92% and 4.90%, respectively, compared with EWP (Table 1). In contrast, aromatic amino acids (AAAs), essential amino acids (EAAs), and total amino acids (TAAs) showed no difference among treatments (*p* > 0.05), although mean TAA concentrations were numerically lower in all enzyme-treated digesta than in EWP.

**Fig. 9:**
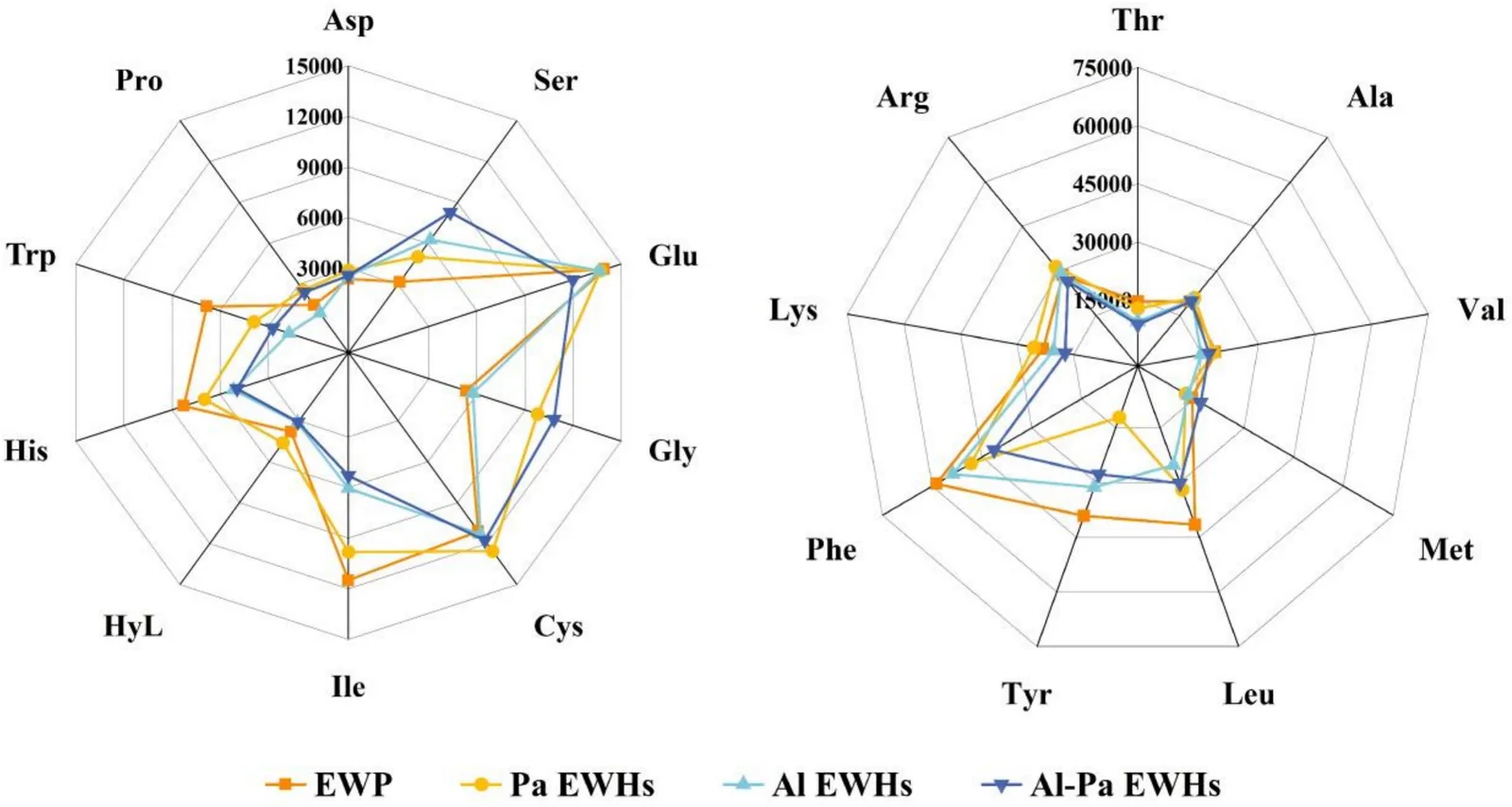
The amino acid composition (ng/mL) of EWP and EWHs. Abbreviation: Al is single-alcalase treatment; Pa is single-papain treatment; Al—Pa is composite alcalase and papain treatment.

**Table 1:** The amino acid composition of different samples (ng/ml).

	EWP	Al	Pa	Al-Pa
HAAs	175.02 ± 17.20 bc	181.28 ± 18.41 ab	169.59 ± 18.56 c	183.61 ± 17.84 a
BCAAs	69.71 ± 12.26 b	74.02 ± 17.70 ab	70.77 ± 7.35 b	79.41 ± 5.36 a
AAAs	104.76 ± 18.29 a	96.81 ± 19.46 a	94.35 ± 13.45 a	91.84 ± 19.77 a
EAAs	239.19 ± 19.42 a	237.59 ± 20.89 a	237.46 ± 19.63 a	239.22 ± 21.07 a
TAAs	355.17 ± 11.57 a	323.90 ± 20.58 a	332.31 ± 29.05 a	335.30 ± 16.33 a

Hydrophobic amino acids (HAAs): Ala, Val, Ile, Leu, Phe, Pro, Met, Cys.

Branch chain amino acids (BCAAs): Leu, Ile, Val.

Aromatic amino acids (AAAs): Phe, Tyr, Trp.

Essential amino acid (EAAs): Thr, Cys, Val, Met, Ile, Leu, Tyr, Phe, Lys, His.

Total amino acids (TAAs).

Abbreviation: Al is single-alcalase treatment; Pa is single-papain treatment; Al—Pa is composite alcalase and papain treatment. Different lowercase letters indicate significant differences between groups (*n* = 3, *p* < 0.05).

These variations may be associated with the distinct cleavage preferences of Al and Pa. Their combined action can produce a different peptide population from that generated by either protease alone, potentially influencing the composition of the products remaining after gastrointestinal digestion (Suwareh et al., 2024; Wang et al., 2025). Together with the higher proportion of low-MW components obtained with Al—Pa (Fig. 2), the differences in the amino acid profiles of the digesta may originate from the distinct peptide compositions in the EWHs. The compositional differences may also be relevant to the antioxidant behavior of the digesta. In particular, the relatively high Cys and Met levels reported for Al—Pa (Fig. 9), along with its higher HAA concentration, may provide a favorable amino acid context for radical-scavenging reactions, given the potential contributions of sulfur-containing and hydrophobic residues (Cui et al., 2025; Wan et al., 2023). These results may be related to the observed stronger DPPH free radical scavenging reaction and the sustained ABTS activity for Al—Pa. However, antioxidant performance also depends on peptide size, sequence, and residue accessibility; therefore, the contribution of individual amino acid groups cannot be established from compositional data alone. Overall, the results suggest that composite hydrolysis is associated with a distinct amino acid profile in the final digesta, which may contribute to its persistent antioxidant functionality.

## 4 Conclusion

This study demonstrates that composite Al—Pa hydrolysis is an effective strategy for EWP hydrolysis in enhancing structural accessibility and sustained antioxidant functionality. Compared with single-protease treatments, the composite protease promoted more extensive protein cleavage, generated a greater proportion of low-MW peptides and induced pronounced conformational rearrangement. These structural modifications indicate that the benefits of composite hydrolysis arise not simply from a higher degree of hydrolysis, but from coordinated changes in peptide size, molecular structure, and chemical composition. Importantly, the Al—Pa hydrolysates maintained strong antioxidant activity during prolonged simulated gastrointestinal digestion. This suggests that the functional characteristics generated during hydrolysis were relatively resistant to subsequent digestive proteolysis. The enrichment of HAAs and BCAAs after digestion further supports the compositional basis of this persistent activity. Collectively, these findings establish a clear structure–composition–function relationship and highlight composite hydrolysis as a promising approach for developing EWP-derived functional ingredients with improved digestive stability. Future work should identify the key bioactive peptide sequences and verify their bioavailability and physiological efficacy in vivo.

## CRediT authorship contribution statement

**Jialei Shi:** Writing – original draft, Validation, Methodology, Formal analysis, Data curation, Conceptualization. **Xianzhen Li:** Writing – review & editing, Supervision, Funding acquisition. **Xunyu Song:** Writing – review & editing, Validation, Methodology, Investigation, Formal analysis, Conceptualization. **Siqi Wang:** Writing – review & editing, Project administration, Methodology, Conceptualization.

## Funding

This research was supported by the Foundation of Liaoning Province Education Administration of China (JYTQN2023114).

## Declaration of competing interest

The authors declare that they have no known competing financial interests or personal relationships that could have appeared to influence the work reported in this paper.

## Data Availability

Data will be made available on request.
